# Variation in Soil Biochemical Properties and Soil Enzyme Activities Among Four Temperate Plantation Types: A Case Study from Beijing, China

**DOI:** 10.3390/plants15213422

**Published:** 2026-11-03

**Authors:** Jinhang Xu, Chenchen Wang, Xixian Kang, Jie Li, Xiangyang Sun

**Affiliations:** The Key Laboratory of Comprehensive Utilization of Green Waste in Hebei Province, College of Forestry, Beijing Forestry University, Beijing 100083, China; xjh923658@163.com (J.X.); yuezongwei_2016@bjfu.edu.cn (C.W.); kang.xixian@foxmail.com (X.K.); jum5892@163.com (J.L.)

**Keywords:** soil nutrients, enzyme activity, plantation type, planted forests, North China

## Abstract

We compared soil biochemical properties in four 11-year-old plantations established under Beijing’s Plain Afforestation Project in the Yanqing–Huailai Basin. The plantations were dominated by *Populus alba*, *Ulmus pumila*, *Salix matsudana*, and *Pinus tabuliformis*, with three plots per plantation. Soil properties were measured at 0–10, 10–30, and 30–60 cm; β-glucosidase (BG), N-acetyl-β-D-glucosaminidase, urease (UE), and dehydrogenase activities were measured at the two upper depths. Two-way mixed-design analysis of variance assessed plantation type, soil depth, and their interaction. Plantation-type effects were significant for available potassium (AK), soil organic carbon (SOC), nitrate (NO_3_^−^), and all four enzyme activities, but not for pH, available phosphorus, or alkali-hydrolyzable nitrogen. Significant interactions occurred for NO_3_^−^, AK, UE, and BG. Mean enzyme activities were higher at 0–10 cm than at 10–30 cm. Pearson analysis of 24 plot-by-depth observations showed positive BG–SOC (r = 0.516) and UE–AK (r = 0.766) associations and a negative UE–NO_3_^−^ association (r = −0.665). Redundancy analysis based on eight plantation-by-depth means identified AK as the only significant explanatory variable, accounting for 41.0% of enzyme activity variation (*p* = 0.018). These findings provide a regional baseline for assessing soil nutrient conditions and selected biochemical activities across plantation types and soil depths.

## 1. Introduction

Planted forests have expanded worldwide and increasingly contribute to wood production, carbon sequestration, land restoration, and other ecosystem services [1]. However, their long-term ecological performance depends not only on climate and silvicultural management but also on the identity of the tree species planted. Tree species differ in litter quantity and quality, which can influence litter decomposition and forest-floor dynamics. They also differ in fine-root traits, nutrient uptake, rhizodeposition, and mycorrhizal associations, which can generate contrasting rhizosphere effects on microbial activity and nutrient availability [2]. Through these pathways, tree species can modify soil organic matter inputs, acidity, and nutrient cycling [3,4,5,6]. Consequently, plantations established under broadly similar environmental conditions may develop contrasting soil biochemical environments, with implications for nutrient retention, carbon storage, and long-term stand sustainability. Evaluating these belowground differences is therefore important for tree species selection and for assessing the ecological outcomes of large-scale afforestation programs.

Soil enzymes mediate organic matter decomposition and nutrient transformation, and their activities are widely used as process-based indicators of soil biochemical functioning [7,8,9]. Hydrolytic enzymes such as cellulases, invertases, and β-glucosidase participate in the degradation of carbon-containing substrates, whereas enzymes involved in nitrogen cycling catalyze the breakdown or transformation of organic nitrogen compounds [10]. Among the enzymes measured in this study, β-glucosidase (BG) releases glucose from cellulose-derived substrates and is commonly used as an indicator of microbial carbon acquisition; N-acetyl-β-D-glucosaminidase (NAG) hydrolyzes chitin-derived compounds; and urease (UE) catalyzes the hydrolysis of urea to ammonia. Dehydrogenase (DHA) differs from these hydrolytic enzymes because it is primarily intracellular and reflects the oxidative activity of viable microbial cells. Taken together, BG, NAG, UE, and DHA provide complementary information on selected processes of carbon decomposition, nitrogen acquisition and transformation, and overall microbial activity. However, because these enzymes do not represent the full suite of enzymes involved in carbon, nitrogen, and phosphorus cycling, conclusions drawn from them should be restricted to the biochemical processes represented by the four measured enzymes.

Since 2012, Beijing has implemented successive large-scale afforestation programs across its plain region, substantially expanding the area of planted forests [11]. These regional efforts form part of China’s broader planted-forest expansion, which has substantially increased planted-forest area and carbon storage during recent decades [12]. This regional afforestation landscape provides an opportunity to evaluate whether commonly planted tree species are associated with different belowground conditions within the same broad climatic setting. We selected plantations dominated by *Populus alba*, *Ulmus pumila*, *Salix matsudana*, and *Pinus tabuliformis* because these species are commonly used in Beijing’s plain-afforestation programs and represent contrasting tree functional types. The first three are deciduous broadleaved species, whereas *P. tabuliformis* is an evergreen conifer. This contrast in leaf habit and associated litter and root characteristics may be accompanied by differences in soil nutrient cycling and microbial activity. Comparing these locally important plantation types therefore provides region-specific evidence for assessing soil biochemical conditions and guiding future plantation management in Beijing.

Although tree-species-associated differences in forest soils have been widely documented, their direction and magnitude vary with environmental and stand conditions. Global analyses indicate that these responses depend on climate, soil fertility, texture, pH, and stand characteristics [13,14]. A recent meta-analysis further showed that responses of SOC quantity and functional fractions differed among forest modification pathways, including plantation replacement and restoration or stand-age sequences, highlighting the importance of the ecological context when interpreting forest soil carbon patterns [15]. Soil nutrients, microbial biomass, enzyme activities, and microbial community composition also vary vertically, so patterns observed in surface soil may not extend to deeper layers [16,17]. These findings highlight the need for regional comparisons that consider both plantation type and soil depth. For plantations established under Beijing’s plain-afforestation programs, integrated evidence on soil nutrient conditions and enzyme activities across locally common plantation types in the Yanqing–Huailai Basin remains limited. The specific contribution of this study is the joint assessment of soil physicochemical properties and four complementary enzyme activities at multiple soil depths in four locally important plantations of similar age within the same study area. This comparison provides a regional baseline for assessing whether differences among the sampled plantations are consistent across soil depths and whether enzyme activities covary with measured soil properties.

Accordingly, this study compared soil pH, soil organic carbon (SOC), available potassium (AK), available phosphorus (AP), alkali-hydrolyzable nitrogen (AN), nitrate (NO_3_^−^), and the activities of β-glucosidase (BG), N-acetyl-β-D-glucosaminidase (NAG), urease (UE), and dehydrogenase (DHA) among plantations dominated by *P. alba* (PA), *U. pumila* (UP), *S. matsudana* (SM), and *P. tabuliformis* (PT). Soil physicochemical properties were examined at depths of 0–10, 10–30, and 30–60 cm, whereas enzyme activities were examined at 0–10 and 10–30 cm. We tested two hypotheses. First, soil nutrient availability and enzyme activities would differ among plantation types, with differences among plantation types being most pronounced in surface soil and enzyme activities being higher at 0–10 cm than at 10–30 cm. Second, variation in enzyme activities would be associated with soil nutrient status, particularly soil C- and N-related properties. Together, these comparisons aim to characterize the soil biochemical conditions of the sampled plantations and identify associations that warrant further investigation. Because each plantation type was represented by one stand, the observed differences are interpreted as plantation-associated patterns within this regional case study and cannot be attributed exclusively to tree species identity.

## 2. Results

### 2.1. Differences in Soil Physicochemical Properties Among Plantation Types

Two-way analysis of variance (ANOVA) showed significant plantation-type effects on NO_3_^−^ (F = 42.60, *p* < 0.001), AK (F = 11.76, *p* = 0.003), and SOC (F = 7.97, *p* = 0.009). Soil depth significantly affected AK (F = 95.36, *p* < 0.001) and SOC (F = 29.86, *p* < 0.001), whereas its overall effect on NO_3_^−^ was not significant (*p* = 0.067). Significant plantation type × soil depth interactions were detected for NO_3_^−^ (F = 5.65, *p* = 0.019) and AK (F = 9.50, *p* = 0.004), but not for SOC (*p* = 0.110). No significant main effects or interactions were detected for pH, AN, or AP (all *p* > 0.05).

**Figure 1:**
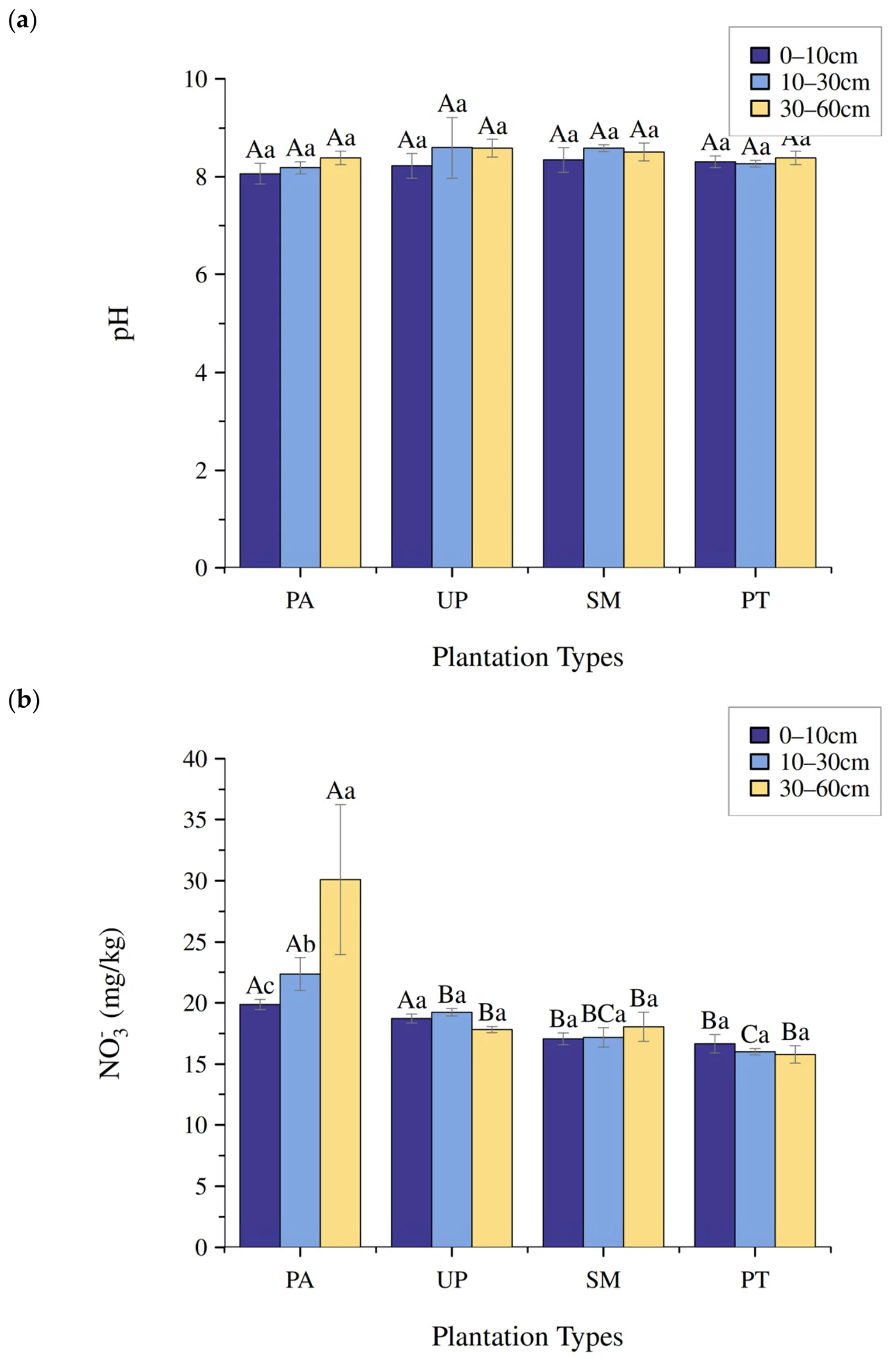

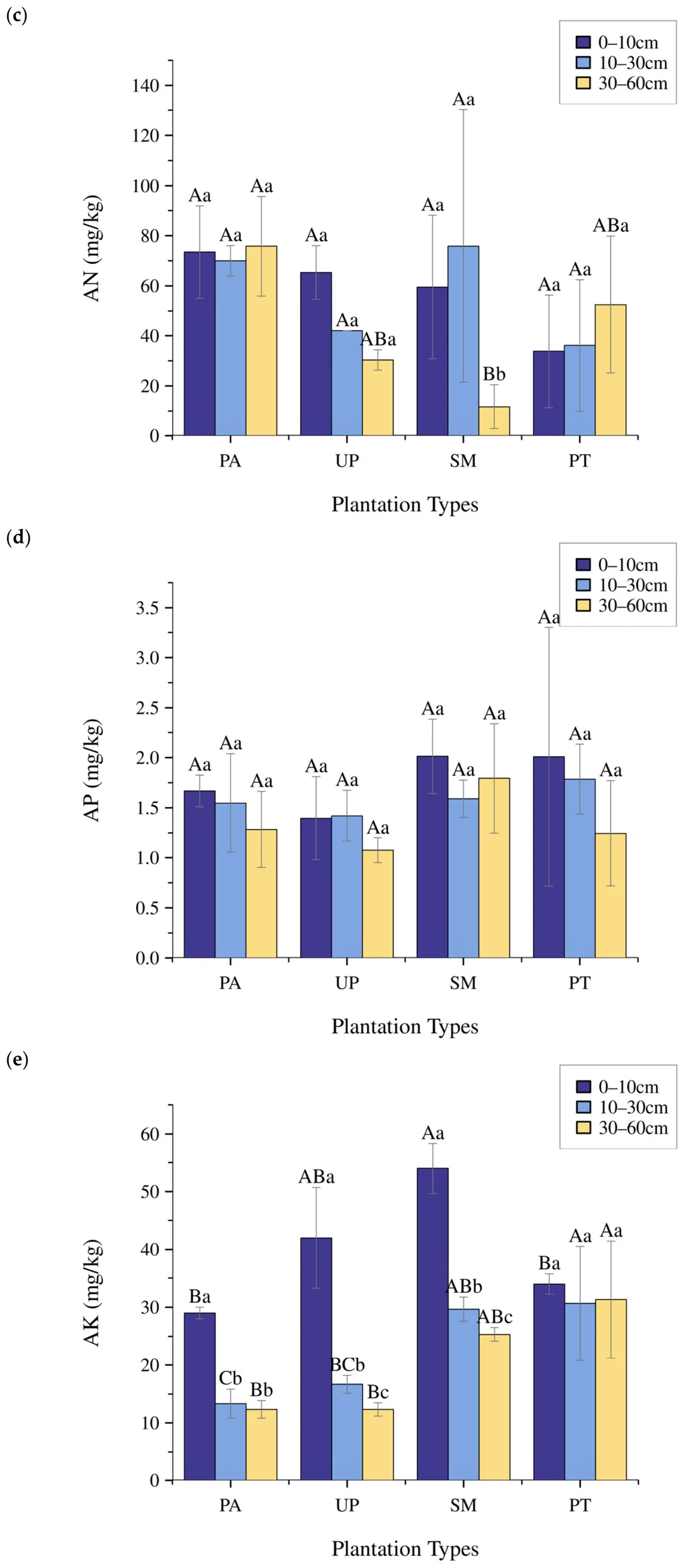

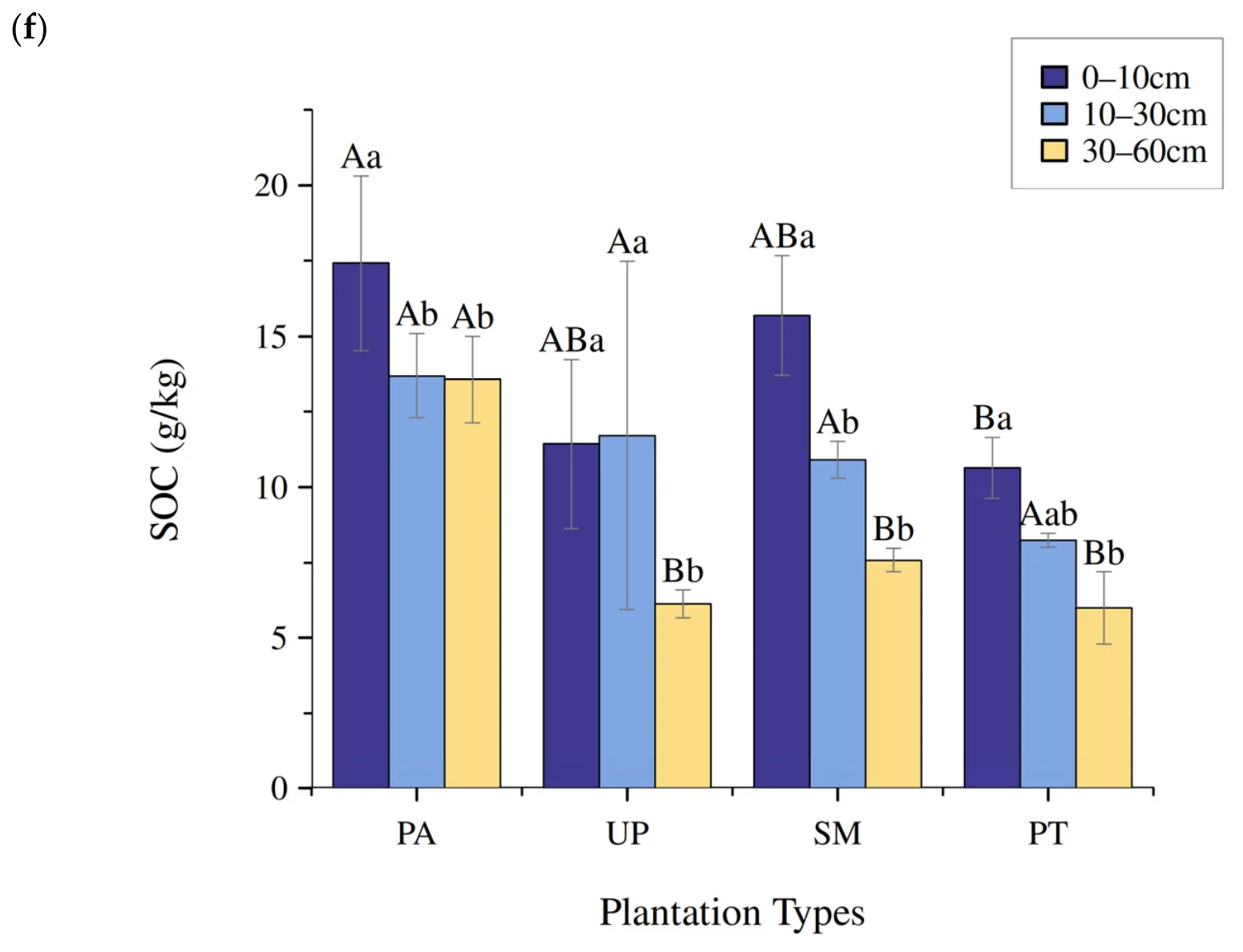
Soil physicochemical properties among four plantation types at three soil depths: (**a**) pH; (**b**) nitrate (NO_3_^−^); (**c**) alkali-hydrolyzable nitrogen (AN); (**d**) available phosphorus (AP); (**e**) available potassium (AK); and (**f**) soil organic carbon (SOC). Bars represent means and error bars indicate standard deviations (*n* = 3 plots per plantation type and soil depth). Uppercase letters indicate comparisons among plantation types within the same soil depth using Tukey’s test. Lowercase letters indicate comparisons among soil depths within the same plantation type using paired contrasts with Holm adjustment. Statistical significance was assessed at adjusted *p* < 0.05. Groups sharing one or more letters are not significantly different within the corresponding comparison.

Mean soil pH ranged from 8.06 to 8.59 across plantation types and soil depths (Figure 1a). Pairwise comparisons showed no significant differences among plantation types within any soil depth or among soil depths within any plantation type.

Mean soil NO_3_^−^ content ranged from 15.79 to 30.09 mg kg^−1^ (Figure 1b). At 0–10 cm, PA and UP had significantly higher NO_3_^−^ contents than SM and PT, with no significant differences between PA and UP or between SM and PT. At 10–30 cm, PA had significantly higher NO_3_^−^ content than the other three plantation types. UP also exceeded PT, whereas SM did not differ significantly from either UP or PT. At 30–60 cm, PA had significantly higher NO_3_^−^ content than the other plantation types, which did not differ significantly from one another. Within PA, NO_3_^−^ increased significantly across successive depths, from 19.86 mg kg^−1^ at 0–10 cm to 22.34 mg kg^−1^ at 10–30 cm and 30.09 mg kg^−1^ at 30–60 cm. No significant depth differences were detected within UP, SM, or PT.

Mean AN content ranged from 11.67 to 75.83 mg kg^−1^ (Figure 1c). At 30–60 cm, AN was significantly higher in PA (75.83 mg kg^−1^) than in SM (11.67 mg kg^−1^). Within SM, AN at 30–60 cm was significantly lower than at both 0–10 and 10–30 cm. The remaining pairwise comparisons for AN were not significant. Mean AP content ranged from 1.07 to 2.01 mg kg^−1^, with no significant pairwise differences among plantation types or soil depths (Figure 1d).

Mean AK content ranged from 12.33 to 54.00 mg kg^−1^ (Figure 1e). At 0–10 cm, SM had significantly higher AK content than PA and PT, whereas UP did not differ significantly from any other plantation type. At 10–30 cm, AK was significantly higher in SM and PT than in PA, and PT also exceeded UP. At 30–60 cm, PT had significantly higher AK content than PA and UP, whereas SM did not differ significantly from the other plantation types. The remaining comparisons among plantation types within each depth were not significant. Within PA, AK was significantly higher at 0–10 cm than at either deeper layer, with no significant difference between 10–30 and 30–60 cm. Within UP and SM, AK decreased significantly across successive depths. No significant depth differences were detected within PT.

Mean SOC content ranged from 5.99 to 17.42 g kg^−1^ (Figure 1f). At 0–10 cm, SOC was significantly higher in PA (17.42 g kg^−1^) than in PT (10.64 g kg^−1^), whereas the other pairwise comparisons were not significant. At 10–30 cm, SOC did not differ significantly among plantation types. At 30–60 cm, PA had significantly higher SOC content (13.57 g kg^−1^) than UP, SM, and PT, whose means ranged from 5.99 to 7.58 g kg^−1^ and did not differ significantly from one another. Within PA and SM, SOC was significantly higher at 0–10 cm than at either deeper layer, with no significant difference between the two deeper layers. Within UP, SOC at 30–60 cm was significantly lower than at both shallower layers. Within PT, only the difference between 0–10 and 30–60 cm was significant.

### 2.2. Differences in Soil Enzyme Activities Among Plantation Types

Two-way ANOVA showed significant plantation-type effects on NAG (F = 4.25, *p* = 0.045), UE (F = 101.64, *p* < 0.001), DHA (F = 43.11, *p* < 0.001), and BG (F = 5.86, *p* = 0.020). Soil depth significantly affected all four enzyme activities: NAG (F = 41.34), UE (F = 53.47), DHA (F = 170.69), and BG (F = 341.86), with all *p* values below 0.001. Significant plantation type × soil depth interactions were detected for UE (F = 4.34, *p* = 0.043) and BG (F = 18.73, *p* < 0.001), whereas the interactions for NAG and DHA were not significant (*p* = 0.246 and 0.143, respectively).

**Figure 2:**
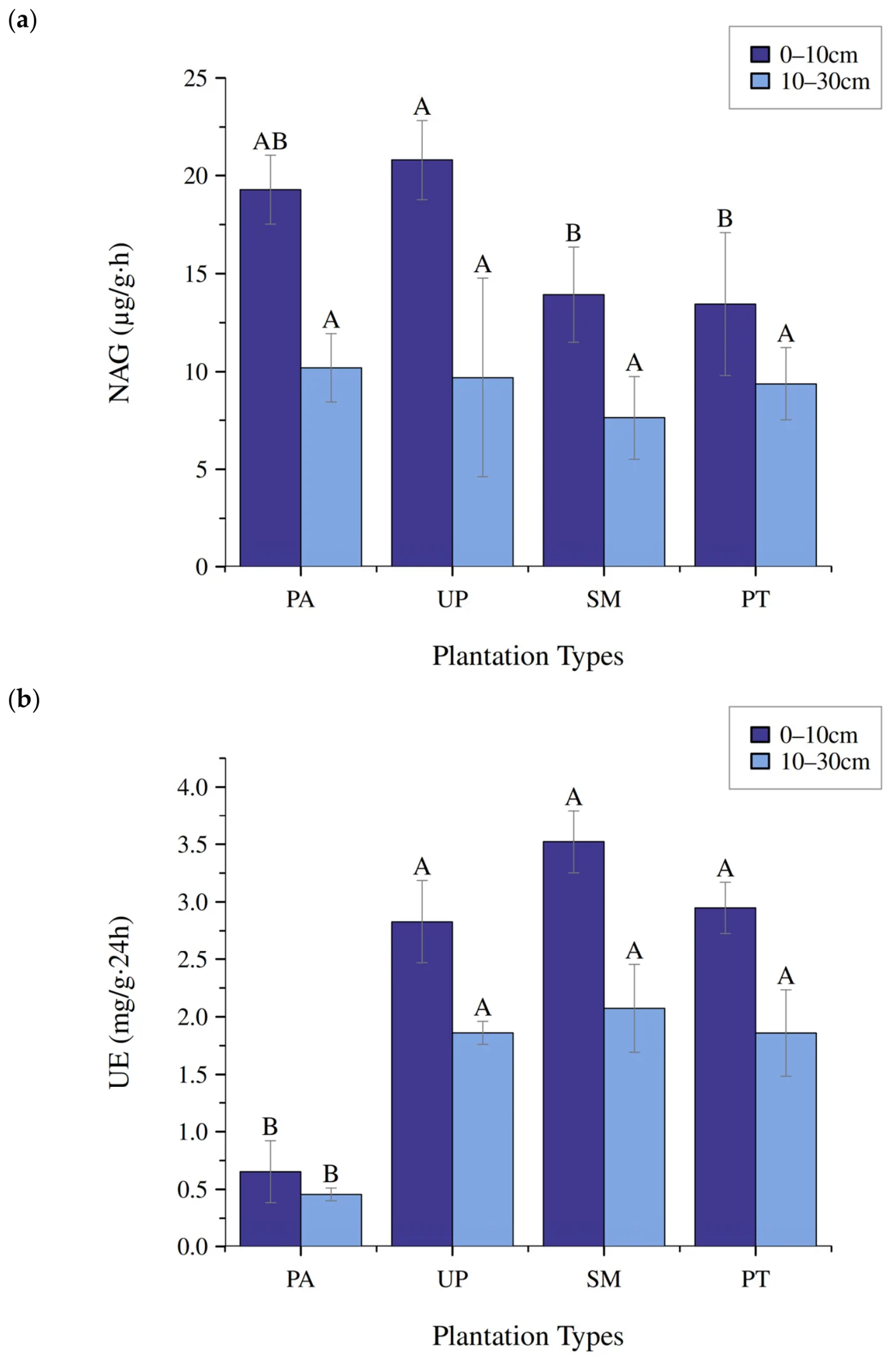

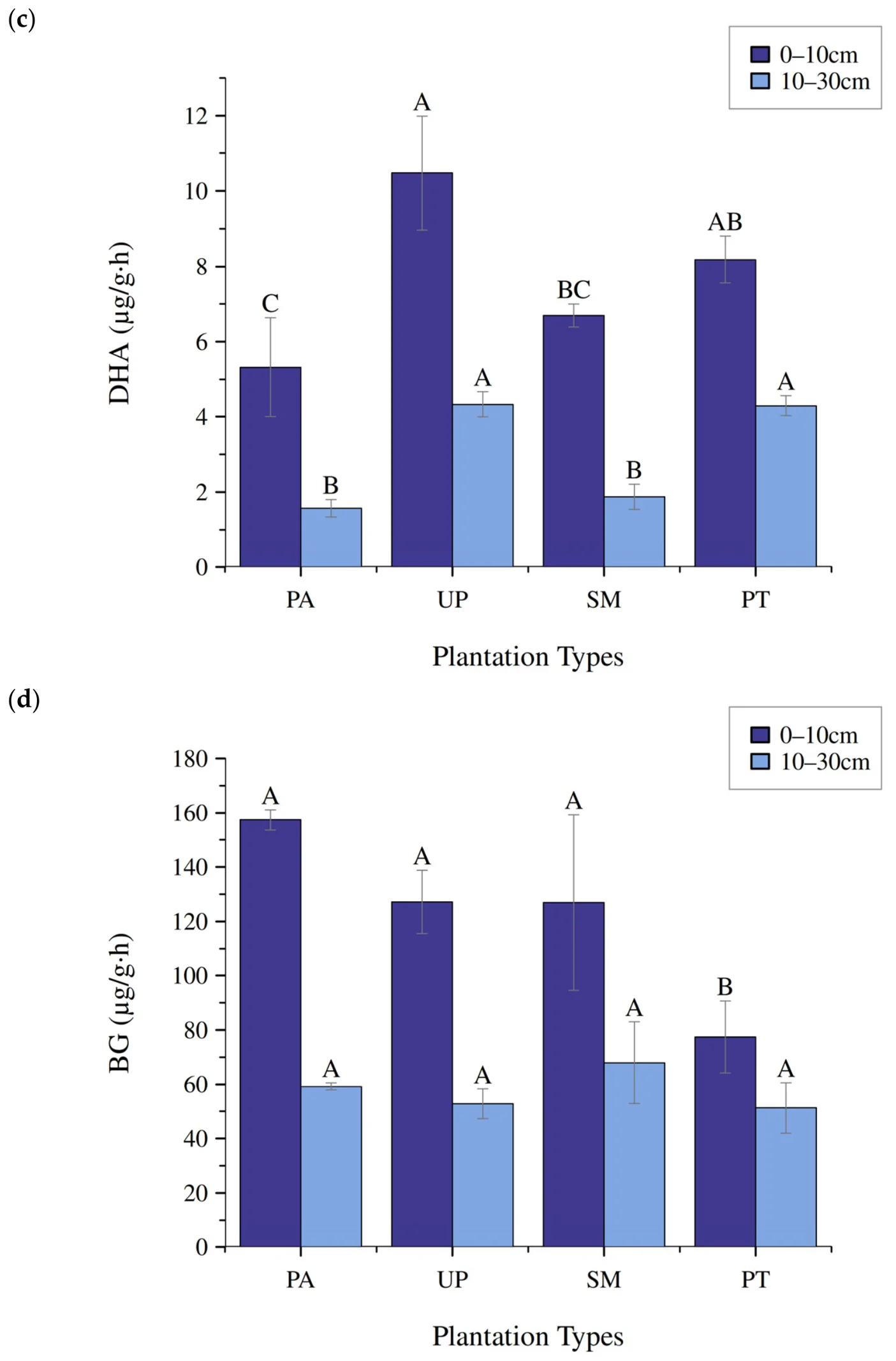
Activities of four soil enzymes among four plantation types at two soil depths: (**a**) N-acetyl-β-D-glucosaminidase (NAG); (**b**) urease (UE); (**c**) dehydrogenase (DHA); and (**d**) β-glucosidase (BG). Bars represent means and error bars indicate standard deviations (*n* = 3 plots per plantation type and soil depth). Uppercase letters indicate pairwise comparisons among plantation types within the same soil depth using Tukey’s test, with significance assessed at adjusted *p* < 0.05. Groups sharing one or more letters are not significantly different within the corresponding comparison.

Mean NAG activity ranged from 7.633 to 20.798 μg pNP g^−1^ h^−1^ (Figure 2a). At 0–10 cm, UP had significantly higher NAG activity (20.798 μg pNP g^−1^ h^−1^) than SM and PT, whose means were 13.928 and 13.449 μg pNP g^−1^ h^−1^, respectively. PA had a mean activity of 19.278 μg pNP g^−1^ h^−1^ and did not differ significantly from any other plantation type. The difference between SM and PT was also not significant. At 10–30 cm, mean NAG activity ranged from 7.633 to 10.201 μg pNP g^−1^ h^−1^, with no significant differences among plantation types.

Mean UE activity ranged from 0.456 to 3.523 mg NH_3_–N g^−1^ d^−1^ (Figure 2b). At both soil depths, PA had significantly lower UE activity than UP, SM, and PT, which did not differ significantly from one another. Mean UE activities in PA were 0.653 and 0.456 mg NH_3_–N g^−1^ d^−1^ at 0–10 and 10–30 cm, respectively. The corresponding means in the other three plantation types ranged from 2.827 to 3.523 mg NH_3_–N g^−1^ d^−1^ at 0–10 cm and from 1.860 to 2.073 mg NH_3_–N g^−1^ d^−1^ at 10–30 cm.

Mean DHA activity ranged from 1.571 to 10.477 μg TPF g^−1^ h^−1^ (Figure 2c). At 0–10 cm, UP had significantly higher DHA activity (10.477 μg TPF g^−1^ h^−1^) than PA and SM, whose means were 5.318 and 6.693 μg TPF g^−1^ h^−1^, respectively. PT also had significantly higher activity (8.183 μg TPF g^−1^ h^−1^) than PA. The remaining pairwise comparisons at this depth were not significant. At 10–30 cm, UP and PT had significantly higher DHA activities, with means of 4.335 and 4.299 μg TPF g^−1^ h^−1^, respectively, than PA and SM, whose means were 1.571 and 1.876 μg TPF g^−1^ h^−1^. Neither the UP–PT comparison nor the PA–SM comparison was significant.

Mean BG activity ranged from 51.274 to 157.348 μg pNP g^−1^ h^−1^ (Figure 2d). At 0–10 cm, PT had significantly lower BG activity (77.435 μg pNP g^−1^ h^−1^) than the other plantation types, whose means ranged from 126.959 to 157.348 μg pNP g^−1^ h^−1^ and did not differ significantly from one another. At 10–30 cm, mean BG activity ranged from 51.274 to 67.919 μg pNP g^−1^ h^−1^, with no significant differences among plantation types.

Across all plantation types, mean enzyme activities were lower at 10–30 cm than at 0–10 cm. Percentage reductions ranged from 30.3% to 53.4% for NAG, from 30.2% to 41.1% for UE, from 47.5% to 72.0% for DHA, and from 33.8% to 62.4% for BG. Paired depth comparisons showed significant reductions in NAG within PA, UP, and SM, and in UE within UP, SM, and PT. The depth differences in NAG within PT and in UE within PA were not significant. DHA and BG activities decreased significantly with depth within all four plantation types.

### 2.3. Relationships Between Soil Properties and Enzyme Activities

**Figure 3:**
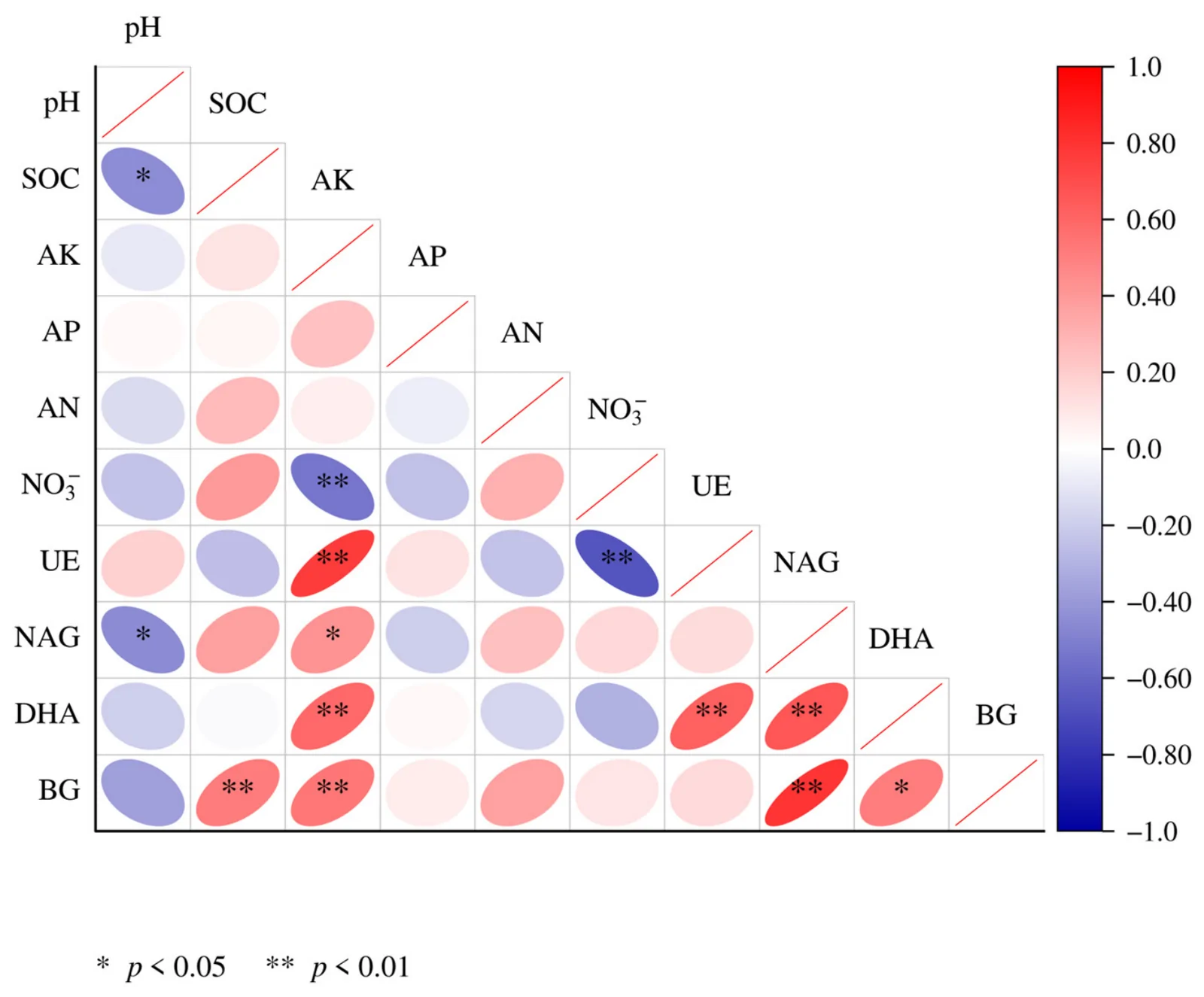
Pearson correlations among soil physicochemical properties and the activities of four soil enzymes, based on 24 matched plot-by-depth samples from the 0–10 and 10–30 cm soil layers. Red and blue ellipses indicate positive and negative correlations, respectively; color intensity and ellipse shape reflect the magnitude of the correlation coefficient. Asterisks indicate statistical significance based on two-sided, unadjusted *p* values (* *p* < 0.05; ** *p* < 0.01). Complete numerical correlation coefficients and corresponding *p* values are provided in Table S1.

Pearson correlation analysis based on 24 plot-by-depth observations showed that UE activity was positively correlated with AK (r = 0.766, *p* < 0.001) and negatively correlated with NO_3_^−^ (r = −0.665, *p* < 0.001; Figure 3). AK was also positively correlated with NAG (r = 0.426, *p* = 0.0378), DHA (r = 0.587, *p* = 0.0026), and BG (r = 0.532, *p* = 0.0074). BG activity was positively correlated with SOC (r = 0.516, *p* = 0.0099), whereas NAG activity was negatively correlated with pH (r = −0.448, *p* = 0.0280).

Among the measured enzymes, NAG activity was positively correlated with BG (r = 0.794, *p* < 0.001) and DHA (r = 0.663, *p* < 0.001). Positive correlations were also observed between UE and DHA (r = 0.623, *p* = 0.0012) and between DHA and BG (r = 0.509, *p* = 0.0110). Complete correlation coefficients and corresponding two-sided *p* values are provided in Table S1.

**Figure 4:**
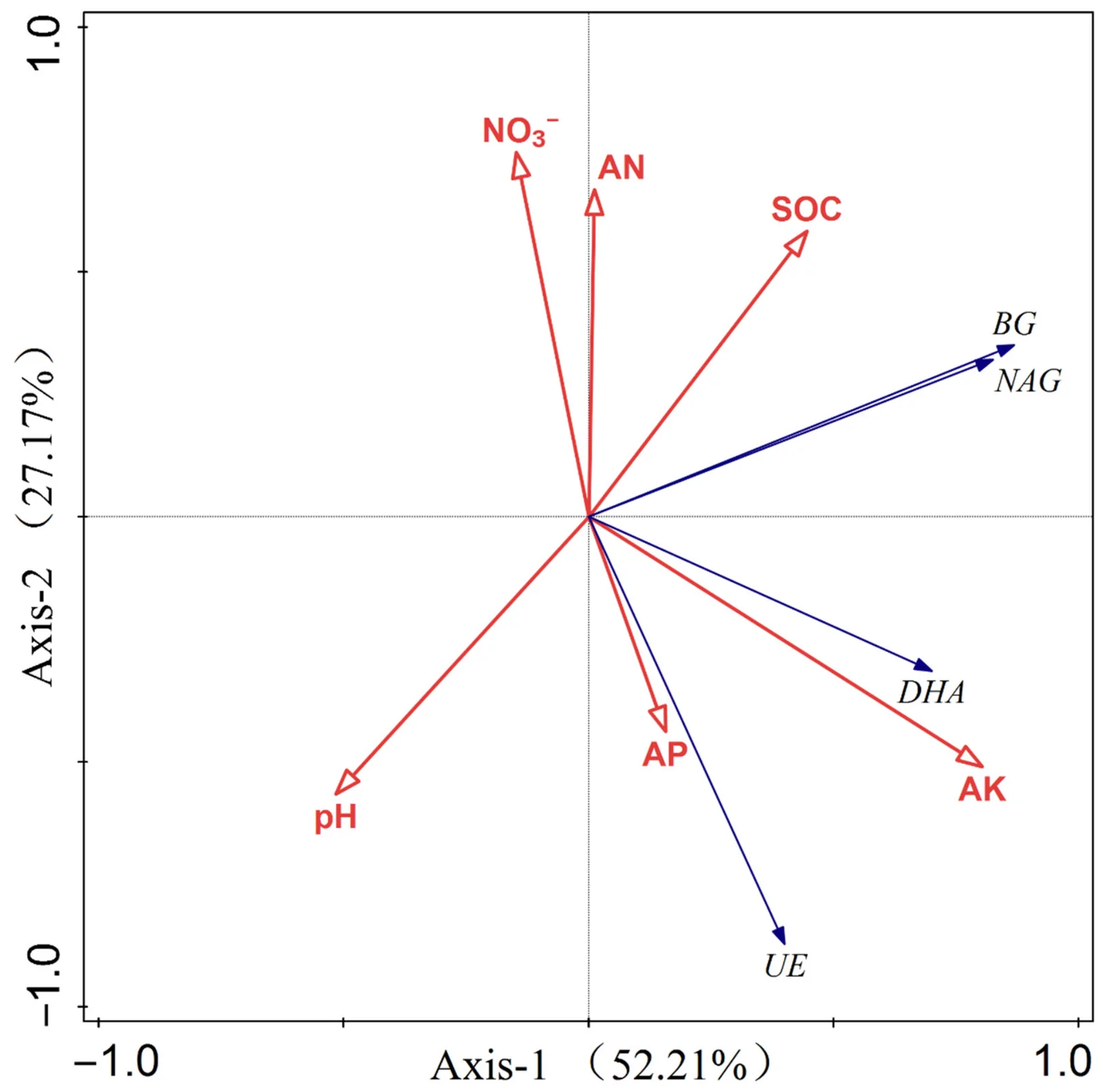
Redundancy analysis of soil physicochemical properties and the activities of the four measured enzymes. Red arrows represent soil physicochemical properties, and blue arrows represent enzyme activities. Arrow length indicates the strength of the association, while the angle between arrows indicates the direction of the relationship.

Redundancy analysis showed that the first two axes explained 79.38% of the variation in the activities of the four measured enzymes (Figure 4). The first axis accounted for 52.21% of the variation, whereas the second axis accounted for 27.17%. The vector orientations were generally consistent with the Pearson correlation results: BG and NAG were oriented toward SOC, DHA was oriented toward AK, and UE was oriented opposite to NO_3_^−^.

The individual effects of the soil properties are presented in Table 1. AK explained 41.0% of the variation and contributed 48.7% of the total explained variation, representing the only statistically significant explanatory variable (pseudo-F = 4.2, *p* = 0.018). Soil pH explained 20.9% and contributed 24.8%, but its effect was not statistically significant (pseudo-F = 2.7, *p* = 0.102). SOC and AP explained 9.3% and 8.0% of the variation, respectively, whereas AN and NO_3_^−^ each explained less than 3%. None of these four variables showed a significant individual effect (*p* > 0.05). These results identify AK as the soil property most strongly associated with variation in the measured enzyme activities, while the apparent influence of pH should be interpreted cautiously.

## 3. Discussion

### 3.1. Plantation-Associated Variation in Soil Physicochemical Properties

The four plantation types exhibited differences in selected soil physicochemical properties. Significant plantation-type effects were detected for AK, SOC, and NO_3_^−^, whereas the overall effects on pH, AP, and AN were not significant. PA generally had the highest mean SOC and NO_3_^−^ contents, while SM had the highest mean AK content in surface soil. These findings partially support our first hypothesis, showing that differences among plantation types were expressed in particular soil properties.

Temperate common-garden and plantation studies have shown that tree species can modify mineral-soil C and N pools [18], as well as soil acidity and base-cation availability through differences in litter chemistry, root inputs, nutrient uptake, and element cycling [5,19,20]. Common-garden evidence also indicates that tree species differ in their contributions to soil C accumulation and N retention [21]. A comparison of 14 tree species further linked soil C stocks to the interacting effects of litter traits, soil biota, and soil chemistry [22]. These pathways provide a framework for interpreting the patterns observed here. Differences in the quantity, composition, and decomposition of litter and root inputs may have contributed to the contrasting SOC and nutrient conditions among the four plantations.

The significant plantation type × soil depth interactions for AK and NO_3_^−^ indicate that their vertical patterns differed among plantation types. AK was enriched in surface soil within PA, UP, and SM, and decreased significantly across successive depths within UP and SM. Such surface enrichment may be associated with nutrient return through litter and biological cycling in the upper soil profile. In PA, however, NO_3_^−^ increased significantly with depth, demonstrating that different nutrients exhibited distinct vertical distributions within the same plantation. SOC showed a significant overall decrease with depth, without a significant plantation type × depth interaction. Although SOC was lower in the deeper layers than in surface soil within PA, its SOC content at 30–60 cm remained significantly higher than that of the other plantation types. PA also maintained the highest NO_3_^−^ content at this depth. These patterns highlight the contribution of deeper soil measurements to characterizing nutrient conditions among the plantations.

The absence of significant pH differences may be associated with the shared soil-forming environment, with soil buffering processes potentially contributing to the relatively similar pH values. The contrasting patterns in AK, SOC, and NO_3_^−^ alongside these similar pH values suggest that nutrient conditions can differ among plantations without a corresponding detectable difference in bulk-soil acidity. Assessing several soil properties across multiple depths therefore provides complementary information for evaluating soil conditions in these plantations.

### 3.2. Associations Between Plantation Types and Soil Enzyme Activities

All four measured enzyme activities varied significantly among plantation types, partially supporting our first hypothesis. The patterns differed among enzymes. In surface soil, BG activity was lower in PT than in the other plantation types, while NAG activity was higher in UP than in SM and PT, with PA showing an intermediate pattern. No significant differences among plantation types were detected for either enzyme at 10–30 cm. UE activity remained lower in PA than in the other three plantation types at both depths, whereas DHA activity at 10–30 cm was higher in UP and PT than in PA and SM. The significant plantation type × soil depth interactions for UE and BG further indicate that the magnitude of their contrasts among plantation types varied with depth.

Mean activities of all four enzymes were lower at 10–30 cm than at 0–10 cm. Reductions ranged from 30.2% to 41.1% for UE, from 33.8% to 62.4% for BG, from 30.3% to 53.4% for NAG, and from 47.5% to 72.0% for DHA. Depth differences were significant for DHA and BG in all four plantation types, for NAG in PA, UP, and SM, and for UE in UP, SM, and PT. These vertical patterns are consistent with the concentration of plant-derived substrates and microbial activity in upper soil layers. Experimental manipulations and quantitative syntheses have shown that litter and root inputs can influence forest-soil enzyme activities [23,24]. Comparisons among forest types have also linked microbial communities and enzyme activities to soil organic matter and moisture conditions [25]. These findings provide plausible explanations for the higher mean enzyme activities observed in surface soil.

Differences among plantation types may also be associated with variation in the quantity and composition of litter and root-derived substrates and in the microbial communities using these resources. Common-garden studies have shown that tree species with contrasting litter characteristics and mycorrhizal associations can support different soil microbial communities and activities, partly through differences in soil C/N ratios and pH [4]. Such variation in plant inputs and microbial resource use may contribute to the distinct patterns observed for BG, NAG, UE, and DHA. The contrasting responses of these enzymes suggest that plantation-associated differences involve several components of soil biochemical functioning.

The four enzymes provide complementary information on these components. Global syntheses have shown that C-, N-, and P-acquiring enzymes and oxidative enzymes can respond differently to environmental drivers [26]. BG reflects the breakdown of cellulose-derived substrates, NAG reflects the hydrolysis of chitin-derived compounds, and UE catalyzes urea hydrolysis. DHA is primarily intracellular and serves as an indicator of microbial oxidative activity. Considering these activities together helps characterize differences in selected substrate transformations and microbial activity among the plantations.

### 3.3. Associations Between Soil Physicochemical Properties and Enzyme Activities

Pearson analysis of the 24 plot-by-depth observations identified associations between soil properties and enzyme activities. BG activity was positively correlated with SOC (r = 0.516, *p* = 0.0099), while UE activity was positively correlated with AK (r = 0.766, *p* < 0.001) and negatively correlated with NO_3_^−^ (r = −0.665, *p* < 0.001). Positive associations of NAG with BG and DHA also indicated that several enzyme activities varied together across the sampled soils. These relationships describe covariation across plantation types and soil depths.

The positive BG–SOC relationship is consistent with the role of BG in the breakdown of cellulose-derived substrates. Higher SOC may coincide with greater supplies of organic substrates supporting microorganisms and their C-acquiring enzymes. Soil enzyme activities are jointly influenced by substrate availability, microbial production, enzyme stabilization, moisture, pH, and other physicochemical conditions [8]. Experimental evidence also shows that extracellular enzyme responses depend on the form and availability of nutrient inputs [27]. The BG–SOC association may therefore reflect linked variation in organic substrates and microbial activity. Similarly, the positive associations of NAG with BG and DHA may reflect shared responses to substrate supply and microbial activity. The negative association between pH and NAG suggests that variation in the soil chemical environment was also associated with this enzyme.

The positive AK–UE relationship should be considered in the context of these shared soil conditions. AK was positively correlated with all four measured enzyme activities, suggesting that its association with UE formed part of a broader pattern of covariation. Both AK and UE had higher mean values at 0–10 cm than at 10–30 cm in all four plantation types, so their common vertical patterns may have contributed to the pooled correlation. Differences among plantation types in nutrient cycling, plant-derived inputs, and microbial activity could also contribute to this association. AK is neither the substrate nor the reaction product of UE, and the correlation does not establish that AK directly activates the enzyme. Its association with UE may instead reflect common responses to soil conditions or other covarying factors.

The negative UE–NO_3_^−^ relationship likewise concerns two different components of soil N transformation. UE catalyzes urea hydrolysis to ammonia, while the soil NO_3_^−^ pool is influenced by nitrification, biological uptake, and nitrate transport. In the present dataset, PA combined relatively high NO_3_^−^ content with low UE activity, and its NO_3_^−^ content increased with depth while mean UE activity decreased. These contrasting patterns may have contributed to the negative correlation. The observed relationship therefore cannot be interpreted as a direct reduction of nitrate availability by UE.

At the level of the eight plantation type × soil depth means, the first two RDA axes explained 52.21% and 27.17% of the variation in enzyme activities, respectively, accounting for 79.38% in total. AK explained 41.0% of the variation and was the only soil property with a significant individual explanatory effect (pseudo-F = 4.2, *p* = 0.018). Soil pH explained 20.9%, although its effect was not significant (*p* = 0.102), and the individual effects of SOC, AP, AN, and NO_3_^−^ were also not significant. The positive BG–SOC and negative UE–NO_3_^−^ relationships provide partial support for our second hypothesis concerning associations with C- and N-related properties. The additional associations with AK highlight the relevance of other soil nutrient conditions when interpreting variation in the measured enzyme activities.

### 3.4. Limitations

This study compared four 11-year-old plantations within a common study area, with three sampling plots established in each stand. This arrangement helped limit broad geographical and stand-age variation. Each plantation type was represented by one continuous stand, so unmeasured stand characteristics may also have contributed to the observed differences. The findings therefore characterize plantation-associated patterns under the local study conditions. Comparisons across additional stands and locations would help establish the consistency of these patterns.

All samples were collected within three days in June 2024, providing a closely timed comparison among the plantations. The findings represent conditions during this summer sampling period; observations at other times of year would extend the assessment to seasonal variation. Soil physicochemical properties were examined to 60 cm, while enzyme measurements focused on four selected activities in the upper 30 cm. Measurements of enzyme activities in deeper layers, together with ammonium and P-acquiring enzymes, would provide additional information on the vertical distribution of biochemical activity, inorganic N availability, and P cycling.

The proposed roles of litter inputs, root traits, microbial properties, and soil moisture remain potential explanations because these variables were not directly measured. Pearson analysis used 24 plot-by-depth observations, including two depths within each plot, whereas RDA used eight plantation type × soil depth means. These analyses summarize associations across plantation types and depths, and shared environmental gradients may contribute to the observed relationships. Future measurements of plant inputs and microbial properties would help evaluate the processes underlying these associations. Bulk density was represented by one core sample per plantation type and soil depth and was used only as descriptive site information, without inclusion in the statistical analyses.

## 4. Materials and Methods

### 4.1. Sampling Sites

The study was conducted in four plantation stands established under Beijing’s Plain Afforestation Project near Fuyutun Village, Yanqing Town, Yanqing District, northwestern Beijing, China (40°28′ N, 115°55′ E; 472 m a.s.l.). The study site is located in the eastern Yanqing–Huailai Basin and has a warm-temperate, semi-humid continental monsoon climate characterized by cold, dry winters and warm, rainy summers. The soil at the study site was classified as a Luvisol according to the World Reference Base for Soil Resources (WRB). All four plantation types were located within the same study area, thereby limiting broad-scale variation in climatic and soil-forming conditions among plantation types.

### 4.2. Experimental Design and Sampling Methods

In June 2024, four 11-year-old monospecific plantation stands were selected within the same study area. The stands were dominated by *Populus alba* (PA), *Ulmus pumila* (UP), *Salix matsudana* (SM), and *Pinus tabuliformis* (PT), respectively. Selecting plantations of the same age within a common geographical setting was intended to limit variation associated with stand age and broad geographical differences when comparing soil conditions among plantation types. Within each stand, three non-overlapping 20 m × 20 m sampling plots were established, with spacing between plots to capture spatial variation within the larger stand, resulting in a total of 12 plots.

All soil samples were collected using a soil auger during a rain-free period within three days in June 2024, minimizing differences in sampling time among the four stands. Within each plot, five sampling points were arranged according to a five-point sampling pattern. Surface vegetation and litter were removed before sampling, and soil was collected separately from depths of 0–10, 10–30, and 30–60 cm. Approximately equal quantities of soil from the five points at each depth were thoroughly combined to form one composite sample per plot and depth. The five sampling points were therefore subsamples contributing to a composite sample. Roots, stones, and other visible debris were removed. This procedure yielded 12 composite samples per soil depth and 36 composite samples in total. Each composite sample was divided into two portions. One portion was air-dried, ground, and passed through 1 mm or 0.149 mm sieves, as appropriate, for the determination of soil physicochemical properties. The other portion was stored at 4 °C for enzyme analysis. Soil enzyme activities were determined in the 0–10 and 10–30 cm samples, yielding 24 composite samples for enzyme analysis. Soil types, textures, and bulk densities across soil depths in the four plantation types are presented in Table 2.

### 4.3. Methods for Determining Soil Physicochemical Properties

Soil bulk density was determined using the core method in accordance with LY/T 1215-1999 [28]. Soil pH was measured potentiometrically in a 1:2.5 soil-to-distilled-water suspension following LY/T 1239-1999 [28]. Soil organic carbon (SOC) was determined using the potassium dichromate oxidation–external heating method according to LY/T 1237-1999 [28]. Available potassium (AK) was extracted with neutral ammonium acetate solution and quantified by flame photometry following LY/T 1234-2015 [28]. Available phosphorus (AP) was extracted with sodium bicarbonate solution and determined using the molybdenum–antimony colorimetric method according to LY/T 1232-2015 [28]. Absorbance was measured at 710 nm using a UV-6100 spectrophotometer(UV-6100, Shanghai Mapada Instruments Co., Ltd., Shanghai, China). Alkali-hydrolyzable nitrogen (AN) was determined using the alkaline hydrolysis diffusion method following LY/T 1228-2015 [28]. Soil nitrate content (NO_3_^−^) was determined by ultraviolet spectrophotometry. All analytical procedures, including reagent concentrations, extraction conditions, calibration, and quality-control requirements, followed the corresponding standards. All reagents used in the analyses were of analytical grade.

### 4.4. Methods for Determining Soil Enzyme Activities

The activities of soil urease (UE), N-acetyl-β-D-glucosaminidase (NAG), dehydrogenase (DHA), and β-glucosidase (BG) were determined in 24 composite soil samples from the 0–10 and 10–30 cm layers. These two layers were selected to compare enzyme activities between surface and subsurface soils within the upper 30 cm of the profile. UE, NAG, and BG were selected to represent urea hydrolysis, the hydrolysis of chitin-derived compounds, and the breakdown of cellulose-derived substrates, respectively. DHA was included as a complementary indicator of microbial oxidative activity. The measurements therefore covered selected processes related to carbon and nitrogen transformation and microbial activity within this depth range. Enzyme activities were not determined at 30–60 cm, and interpretations of enzyme patterns were restricted to the two measured layers. Fresh soil samples stored at 4 °C were submitted to a commercial analytical laboratory (Convinced-Test, Nanjing, China). Before analysis, the samples were pretreated and passed through a 30–50-mesh sieve according to the manufacturer’s instructions. Enzyme activities were determined using commercial assay kits manufactured by Nanjing Mofan Biotechnology Co., Ltd. (Nanjing, China). Soil mass was measured using an ML-204 analytical balance (ML204, Mettler-Toledo AG, Greifensee, Switzerland). Incubations were conducted in an HH-6 thermostatic water bath (HH-6, Guohua (Changzhou) Instrument Manufacturing Co., Ltd., Changzhou, China), samples were centrifuged using a TGL-20bR centrifuge (TGL-20bR, Shanghai Anting Scientific Instrument Factory, Shanghai, China), and absorbance was measured using a SpectraMax 190 microplate reader (SpectraMax 190, Molecular Devices, LLC, San Jose, CA, USA). Sample blanks and calibration standards were included in all assays.

For the UE assay, 0.10 g of pretreated soil was mixed with 100 μL of toluene and allowed to stand for 15 min. Urea substrate solution and the corresponding kit reagent were then added, and the mixture was incubated at 37 °C for 24 h. After centrifugation at 10,000 rpm for 10 min, ammonia in the supernatant was quantified using the phenol–sodium hypochlorite colorimetric method at 630 nm. UE activity was expressed as mg NH_3_–N g^−1^ dry soil d^−1^ [29]. For the NAG assay, 0.10 g of soil was incubated with p-nitrophenyl N-acetyl-β-D-glucosaminide at 37 °C for 3 h. Following centrifugation at 10,000 rpm for 10 min, the released p-nitrophenol (pNP) was quantified at 400 nm. NAG activity was expressed as μg p-nitrophenol g^−1^ dry soil h^−1^ [30].

For the DHA assay, 0.10 g of soil was incubated with 2,3,5-triphenyltetrazolium chloride in darkness at 37 °C for 6 h. The reaction was stopped by cooling the samples in an ice bath, and the resulting triphenyl formazan (TPF) was extracted with acetone. After centrifugation at 15,000 rpm and 4 °C for 10 min, absorbance was measured at 485 nm. DHA activity was expressed as μg triphenyl formazan g^−1^ dry soil h^−1^. For the BG assay, 0.10 g of soil was incubated with p-nitrophenyl-β-D-glucopyranoside at 37 °C for 3 h. The reaction was terminated by heating in boiling water for 5 min. After centrifugation at 10,000 rpm for 10 min, the released p-nitrophenol was quantified at 400 nm. BG activity was expressed as μg p-nitrophenol g^−1^ dry soil h^−1^ [31].

### 4.5. Data Analysis

Data were organized using Microsoft Excel 2021 and are presented as means ± standard deviations based on three plots per plantation type and soil depth. Each response variable was analyzed using a two-way mixed-design analysis of variance (ANOVA), with plantation type as the between-plot factor and soil depth as the within-plot factor. Plots were nested within plantation type and treated as the subjects for repeated measurements across depths. Each model included plantation type, soil depth, and their interaction. Soil physicochemical properties were analyzed across three depths, whereas enzyme activities were analyzed across two depths. For the three-depth analyses, the Greenhouse–Geisser correction was applied to the degrees of freedom for the depth and interaction tests. Residual normality and variance homogeneity were examined using the Shapiro–Wilk and Levene tests, respectively.

For each response variable, pairwise comparisons among plantation types within each soil depth were conducted using Tukey’s honestly significant difference test. Comparisons among soil depths within each plantation type used paired contrasts calculated from the pooled covariance of the plot depth profiles. The resulting *p* values were adjusted using the Holm procedure within each set of depth comparisons for a given plantation type and response variable. Statistical significance was assessed at *p* < 0.05, using adjusted *p* values for the pairwise comparisons.

Pearson correlation analysis was performed using the 24 plot-by-depth observations from four plantation types, three plots per plantation type, and two soil depths (0–10 and 10–30 cm). Soil properties and enzyme activities were matched by plot and depth, without averaging the three plots before analysis. Pearson correlation coefficients and conventional two-sided *p* values were calculated without adjustment for multiple testing. The complete correlation coefficients and corresponding *p* values are provided in Table S1.

Redundancy analysis (RDA) was conducted in Canoco 5 using eight plantation type × soil depth means, obtained by averaging the three plots for each plantation type at each of the two depths examined for enzyme activities. Enzyme activities were treated as response variables and soil physicochemical properties as explanatory variables. The significance of individual explanatory variables was evaluated using the permutation tests implemented in Canoco 5. ANOVA, pairwise comparisons, and Pearson correlation analyses were performed in R version 4.3.3. Figure 1, Figure 2 and Figure 3 were generated using R.

## 5. Conclusions

This regional case study identified distinct soil nutrient and enzyme activity patterns among four 11-year-old plantation types in the Yanqing–Huailai Basin, Beijing. The first hypothesis was partially supported: significant plantation-type effects were detected for AK, SOC, NO_3_^−^, and all four measured enzyme activities, while the overall effects on pH, AP, and AN were not significant. Soil depth significantly affected AK, SOC, and all four enzyme activities. Mean enzyme activities were higher at 0–10 cm than at 10–30 cm across the four plantation types. Significant plantation type × soil depth interactions for NO_3_^−^, AK, UE, and BG further showed that some contrasts among plantations depended on soil depth.

The second hypothesis was also partially supported. BG activity was positively associated with SOC, while UE activity was negatively associated with NO_3_^−^ and positively associated with AK. AK was also positively associated with the other three enzyme activities and was the only soil property with a significant individual explanatory effect in the RDA, accounting for 41.0% of the variation in enzyme activities. These associations highlight links between selected soil biochemical activities and several aspects of soil nutrient conditions. The study provides a regional baseline for assessing soil conditions in locally important plantations and highlights the value of examining nutrient profiles and complementary enzyme activities at multiple soil depths.

## Figures and Tables

**Table 1:** Contributions of soil physicochemical variables to variation in the activities of the four measured soil enzymes based on RDA.

Name	Explains %	Contribution %	Pseudo-F	*p*
AK	41	48.7	4.2	0.018
pH	20.9	24.8	2.7	0.102
SOC	9.3	11.1	1.3	0.352
AP	8	9.5	1.2	0.38
AN	2.7	3.2	0.3	0.774
NO_3_^−^	2.4	2.8	0.2	0.858

**Table 2:** Soil types, textures, and bulk densities across soil depths in the four plantation types.

Plantation Type	Soil Layer	Soil Type	Soil Texture	Soil Bulk Density (g/cm^3^)
*Populus alba*	0–10 cm	Luvisol	Loam	1.32
	10–30 cm	Luvisol	Loam	1.47
	30–60 cm	Luvisol	Loam	1.51
*Ulmus pumila*	0–10 cm	Luvisol	Loam	1.52
	10–30 cm	Luvisol	Loam	1.51
	30–60 cm	Luvisol	Loam	1.57
*Salix matsudana*	0–10 cm	Luvisol	Loam	1.52
	10–30 cm	Luvisol	Loam	1.61
	30–60 cm	Luvisol	Loam	1.66
*Pinus tabuliformis*	0–10 cm	Luvisol	Loam	1.32
	10–30 cm	Luvisol	Loam	1.43
	30–60 cm	Luvisol	Loam	1.45

## Data Availability

The data that support the findings of this study are available from the corresponding author upon reasonable request.

## Supplementary Materials

The following supporting information can be downloaded at: https://www.mdpi.com/article/10.3390/plants15213422/s1, Table S1: Pearson correlations among soil physicochemical properties and enzyme activities across the 24 soil samples.

## References

[1] Payn T., Carnus J.-M., Freer-Smith P., Kimberley M., Kollert W., Liu S., Orazio C., Rodriguez L., Silva L.N., Wingfield M.J. (2015). Changes in Planted Forests and Future Global Implications. For. Ecol. Manag..

[2] Phillips R.P., Fahey T.J. (2006). Tree Species and Mycorrhizal Associations Influence the Magnitude of Rhizosphere Effects. Ecology.

[3] Vesterdal L., Clarke N., Sigurdsson B.D., Gundersen P. (2013). Do Tree Species Influence Soil Carbon Stocks in Temperate and Boreal Forests?. For. Ecol. Manag..

[4] Heděnec P., Nilsson L.O., Zheng H., Gundersen P., Schmidt I.K., Rousk J., Vesterdal L. (2020). Mycorrhizal Association of Common European Tree Species Shapes Biomass and Metabolic Activity of Bacterial and Fungal Communities in Soil. Soil Biol. Biochem..

[5] Mueller K.E., Eissenstat D.M., Hobbie S.E., Oleksyn J., Jagodzinski A.M., Reich P.B., Chadwick O.A., Chorover J. (2012). Tree Species Effects on Coupled Cycles of Carbon, Nitrogen, and Acidity in Mineral Soils at a Common Garden Experiment. Biogeochemistry.

[6] Hobbie S.E., Reich P.B., Oleksyn J., Ogdahl M., Zytkowiak R., Hale C., Karolewski P. (2006). Tree Species Effects on Decomposition and Forest Floor Dynamics in a Common Garden. Ecology.

[7] Nannipieri P., Giagnoni L., Renella G., Puglisi E., Ceccanti B., Masciandaro G., Fornasier F., Moscatelli M.C., Marinari S. (2012). Soil Enzymology: Classical and Molecular Approaches. Biol. Fertil. Soils.

[8] Burns R.G., DeForest J.L., Marxsen J., Sinsabaugh R.L., Stromberger M.E., Wallenstein M.D., Weintraub M.N., Zoppini A. (2013). Soil Enzymes in a Changing Environment: Current Knowledge and Future Directions. Soil Biol. Biochem..

[9] Trasar-Cepeda C., Leiros M.C., Gil-Sotres F. (2008). Hydrolytic Enzyme Activities in Agricultural and Forest Soils. Some Implications for Their Use as Indicators of Soil Quality. Soil Biol. Biochem..

[10] Sinsabaugh R.L., Hill B.H., Follstad Shah J.J. (2009). Ecoenzymatic Stoichiometry of Microbial Organic Nutrient Acquisition in Soil and Sediment. Nature.

[11] Kang X., Li S., Sun X., Wang C., Li J., Xu J. (2025). Patterns of Soil Microbial Residue Carbon Accumulation in Different Plantation Forest Types: A Case Study from Beijing. Forests.

[12] Cheng K., Yang H., Tao S., Su Y., Guan H., Ren Y., Hu T., Li W., Xu G., Chen M. (2024). Carbon Storage through China’s Planted Forest Expansion. Nat. Commun..

[13] Augusto L., Boča A. (2022). Tree Functional Traits, Forest Biomass, and Tree Species Diversity Interact with Site Properties to Drive Forest Soil Carbon. Nat. Commun..

[14] Peng Y., Schmidt I.K., Zheng H., Heděnec P., Bachega L.R., Yue K., Wu F., Vesterdal L. (2020). Tree Species Effects on Topsoil Carbon Stock and Concentration Are Mediated by Tree Species Type, Mycorrhizal Association, and N-Fixing Ability at the Global Scale. For. Ecol. Manag..

[15] Zong J., Sun X., Wang C. (2026). Contrasting Responses of Soil Organic Carbon and Functional Fractions across Forest Modification Pathways: A Meta-Analysis. CATENA.

[16] Stone M.M., DeForest J.L., Plante A.F. (2014). Changes in Extracellular Enzyme Activity and Microbial Community Structure with Soil Depth at the Luquillo Critical Zone Observatory. Soil Biol. Biochem..

[17] Fierer N., Schimel J.P., Holden P.A. (2003). Variations in Microbial Community Composition through Two Soil Depth Profiles. Soil Biol. Biochem..

[18] Finzi A.C., Van Breemen N., Canham C.D. (1998). Canopy Tree–Soil Interactions Within Temperate Forests: Species Effects on Soil Carbon and Nitrogen. Ecol. Appl..

[19] Reich P.B., Oleksyn J., Modrzynski J., Mrozinski P., Hobbie S.E., Eissenstat D.M., Chorover J., Chadwick O.A., Hale C.M., Tjoelker M.G. (2005). Linking Litter Calcium, Earthworms and Soil Properties: A Common Garden Test with 14 Tree Species. Ecol. Lett..

[20] Hagen-Thorn A., Callesen I., Armolaitis K., Nihlgård B. (2004). The Impact of Six European Tree Species on the Chemistry of Mineral Topsoil in Forest Plantations on Former Agricultural Land. For. Ecol. Manag..

[21] Gurmesa G.A., Schmidt I.K., Gundersen P., Vesterdal L. (2013). Soil Carbon Accumulation and Nitrogen Retention Traits of Four Tree Species Grown in Common Gardens. For. Ecol. Manag..

[22] Mueller K.E., Hobbie S.E., Chorover J., Reich P.B., Eisenhauer N., Castellano M.J., Chadwick O.A., Dobies T., Hale C.M., Jagodziński A.M. (2015). Effects of Litter Traits, Soil Biota, and Soil Chemistry on Soil Carbon Stocks at a Common Garden with 14 Tree Species. Biogeochemistry.

[23] Fekete I., Varga C., Kotroczó Z., Tóth J.A., Várbíró G. (2011). The Relation between Various Detritus Inputs and Soil Enzyme Activities in a Central European Deciduous Forest. Geoderma.

[24] Ai L., Wu F., Fan X., Yang Y., Zhang Y., Zheng X., Zhu J., Ni X. (2023). Different Effects of Litter and Root Inputs on Soil Enzyme Activities in Terrestrial Ecosystems. Appl. Soil Ecol..

[25] Brockett B.F.T., Prescott C.E., Grayston S.J. (2012). Soil Moisture Is the Major Factor Influencing Microbial Community Structure and Enzyme Activities across Seven Biogeoclimatic Zones in Western Canada. Soil Biol. Biochem..

[26] Xiao W., Chen X., Jing X., Zhu B. (2018). Meta-Analysis of Soil Extracellular Enzyme Activities in Response to Global Change. Soil Biol. Biochem..

[27] Allison S.D., Vitousek P.M. (2005). Responses of Extracellular Enzymes to Simple and Complex Nutrient Inputs. Soil Biol. Biochem..

[28] Bao S.D. (2000). Soil and Agricultural Chemistry Analysis.

[29] Kandeler E., Gerber H. (1988). Short-Term Assay of Soil Urease Activity Using Colorimetric Determination of Ammonium. Biol. Fertil. Soils.

[30] Parham J.A., Deng S.P. (2000). Detection, Quantification and Characterization of β-Glucosaminidase Activity in Soil. Soil Biol. Biochem..

[31] Eivazi F., Tabatabai M.A. (1988). Glucosidases and Galactosidases in Soils. Soil Biol. Biochem..

