# Supplementary material for: Variation in Soil Biochemical Properties and Soil Enzyme Activities Among Four Temperate Plantation Types: A Case Study from Beijing, China
Source: Plants (Basel). 2026 Nov 3;15(21):3422. doi: 10.3390/plants15213422 (PMC13765903; doi:10.3390/plants15213422)
Supplement: Supplementary file 1 [file plants-15-03422-s001.zip › plants-4611426-supplementary.pdf]

Supplementary material

Table S1. Pearson correlations among soil physicochemical properties and enzyme activities across the 24 soil samples.

Variable	pH	SOC	AK	AP	AN	NO_3^-	UE	NAG	DHA	BG
pH	1.000									
SOC	−0.446* (0.0291)	1.000								
AK	−0.089 (0.6784)	0.105 (0.6246)	1.000							
AP	0.023 (0.9166)	0.038 (0.8605)	0.241 (0.2561)	1.000						
AN	−0.144 (0.5012)	0.268 (0.2054)	0.068 (0.7527)	−0.072 (0.7373)	1.000					
NO_3^-	−0.235 (0.2686)	0.396 (0.0552)	−0.539** (0.0066)	−0.243 (0.2518)	0.308 (0.1429)	1.000				
UE	0.179 (0.4021)	−0.254 (0.2304)	0.766** (1.30×10^{-5})	0.116 (0.5894)	−0.234 (0.2702)	−0.665** (0.0004)	1.000			
NAG	−0.448* (0.0280)	0.371 (0.0744)	0.426* (0.0378)	−0.195 (0.3610)	0.245 (0.2481)	0.151 (0.4816)	0.138 (0.5209)	1.000		
DHA	−0.192 (0.3687)	−0.021 (0.9231)	0.587** (0.0026)	0.030 (0.8910)	−0.163 (0.4453)	−0.307 (0.1443)	0.623** (0.0012)	0.663** (0.0004)	1.000	
BG	−0.374 (0.0714)	0.516** (0.0099)	0.532** (0.0074)	0.072 (0.7398)	0.366 (0.0784)	0.101 (0.6387)	0.145 (0.4993)	0.794** (3.55×10^{-6})	0.509* (0.0110)	1.000

Values are Pearson correlation coefficients (r), with conventional two-sided, unadjusted P values in parentheses. Each correlation used 24 matched samples from four sampled stands, three plots per stand and two soil depths (0–10 and 10–30 cm). * $P < 0.05$; ** $P < 0.01$. The symmetric upper triangle is omitted. Abbreviations: SOC, soil organic carbon; AK, available potassium; AP, available phosphorus; AN, alkali-hydrolyzable nitrogen; NO_3^-, nitrate; UE, urease; NAG, N-acetyl-β-D-glucosaminidase; DHA, dehydrogenase; BG, β-glucosidase.
